# Differentiating trait pain from state pain: a window into brain mechanisms underlying how we experience and cope with pain

**DOI:** 10.1097/PR9.0000000000000735

**Published:** 2019-08-07

**Authors:** Karen D. Davis, Joshua C. Cheng

**Affiliations:** a Department of Surgery and Institute of Medical Science, University of Toronto, Toronto, ON, Canada; b Krembil Brain Institute, Krembil Research Institute, Toronto Western Hospital, University Health Network, Toronto, ON, Canada; c Stony Brook University School of Medicine, Stony Brook, NY, USA

**Keywords:** Brain imaging, fMRI, Connectivity, Behaviour, Trait

## Abstract

Across various biological and psychological attributes, individuals have a set point around which they can fluctuate transiently into various states. However, if one remains in a different state other than their set point for a considerable period (eg, induced by a disease), this different state can be considered to be a new set point that also has associated surrounding states. This concept is instructive for understanding chronic pain, where an individual's set point may maladaptively shift such that they become stuck at a new set point of pain (trait pain), from which pain can fluctuate on different timescales (ie, pain states). Here, we discuss the importance of considering trait and state pains in neuroimaging studies of brain structure and function to gain an understanding of not only an individual's current pain state but also more broadly to their trait pain, which may be more reflective of their general condition.

## 1. Introduction

As pain brain imagers, we seek to understand how pain and pain-related feelings are represented in the brain. We also strive to someday use this information to alleviate pain. However, since the introduction of neuroimaging modalities, there remain many challenges to translate the findings of experimental studies into effective therapies for all who suffer from chronic pain. Here, we will consider just the single issue of linking pain experience with attributes of brain structure and function.

## 2. Set points: states and traits

**Figure 1.**
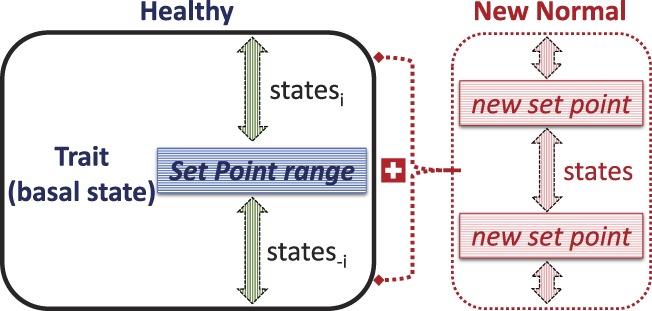
Set point concept. A schematic representation of a “set point concept” to describe biological and psychological attributes of an individual. In a healthy condition, an individual trait (ie, a basal state) is represented by a set point. This set point is not necessarily a “point” but a small range of values. Deviations from this set point range can occur into higher or lower “states” as conditions change transiently. However, after an injury or in disease/pathological conditions, the set point may change and represent a “new normal” trait, from which further deviations to different states are possible.

We can think of an individual as having a “set point” across a variety of biological and psychological attributes (Fig. 1). This can be thought of as a basal or baseline level of who we are, from which we can deviate depending on situational conditions. For example, our weight, general temperament, abilities, and such tend to be characteristic of our essential self. These attributes can also fluctuate according to particular situations, conditions, our efforts, and desires. Psychologists and psychiatrists have long recognized that behaviours and personalities may be transient or an intrinsic attribute and thus developed questionnaires to assess both states and traits (eg, the state-trait anxiety index^38^). The distinction between a condition or situational state vs a characteristic trait is useful to diagnose and treat mental health conditions such as anxiety and depression.

Conceptually, a set point can be thought of as a *trait* with fluctuations that take us to different *states*. Both the trait set point and states likely comprise a small range of values rather than 1 precise “point.” Typically, under normal everyday situations, moving to a different state would be transient, soon to return to the original trait set point. But if time in a different state were to persist, eg, after an injury or a disease progression, this state could represent a “new normal” or set point (Fig. 1). Fluctuations into different states would then revolve around that new rather than original set point. This concept of set points can also be instructive in thinking about acute pain sensations and pain-evoked reactions that are experienced in a healthy individual vs chronic pain. For example, an ability to adapt or cope with situations may be conceptualized as an attempt to return to our set point, and getting there could be impacted by our ability to change. This can be thought of as our capacity for plasticity. Given that plasticity can be adaptive or maladaptive, in this “set point concept,” plasticity would represent moving towards or away from a “good set point.”

As an illustrative example, imagine that an individual has been suffering from chronic pain for many years. Compared with their previous healthy selves, their set point may now have maladaptively shifted into one where they generally experience a certain level of pain. In addition, they may have fluctuations in their chronic pain due to activity, sleep patterns, attentional focus, etc, and these represent their different chronic pain states. Interestingly, some chronic pains show circadian rhythmicity with higher pain being reported in the evenings than mornings or afternoons.^19,20,32^ This needs to be considered when collecting brain imaging and behavioural data because at the time of investigation, they may be experiencing much more or much less pain than they may have on average. Brain imagers have all experienced such situations in which a research participant with chronic pain who meets their inclusion criteria (say, eg, a rating of average pain of a 4/10), arrives at the scanner with either very little or no pain at all, or conversely with having a “bad day” with pain much higher than their usual. Thus, people with chronic pain can exhibit not only a new normal (set point) that could represent their new “trait” but also, additionally, they could move from that point to other states. This idea of concurrent state and trait attributes has been recognized in the field of psychology. For example, a study of the Big-Five behaviours measured within an individual over several weeks revealed that these behaviors fall within a distribution, with stable mean and variance parameters.^16^ Although the central tendency and variance of these behavioral distributions captured trait-like attributes of individuals, the presence of a wide array of possible states within the distribution reflected state-like attributes of the individual. So, given these properties, how should we link individuals' brain data to their pain? Below, we consider the factors of linking brain data to ratings of pain states and trait.

## 3. Pain trait and pain states

What comprises a “pain trait”? This can simply be thought of someone's typical pain response. Behaviourally, we can characterize a person's reaction to painful stimuli and their pain sensitivity based on their response to a battery of psychophysical measures that quantify threshold and suprathreshold responses and tolerance to experimental stimuli. It is generally assumed that these tests give some insight into an individual's intrinsic sensitivity—ie, trait pain—and that this would be more or less stable over time. However, it is also well known that many factors can modify pain sensitivity, such as attention, arousal, and mood.^5,41^ Thus, pain sensitivity could also be deemed a pain state (dependent on the conditions of the test and individual being tested).

**Figure 2.**
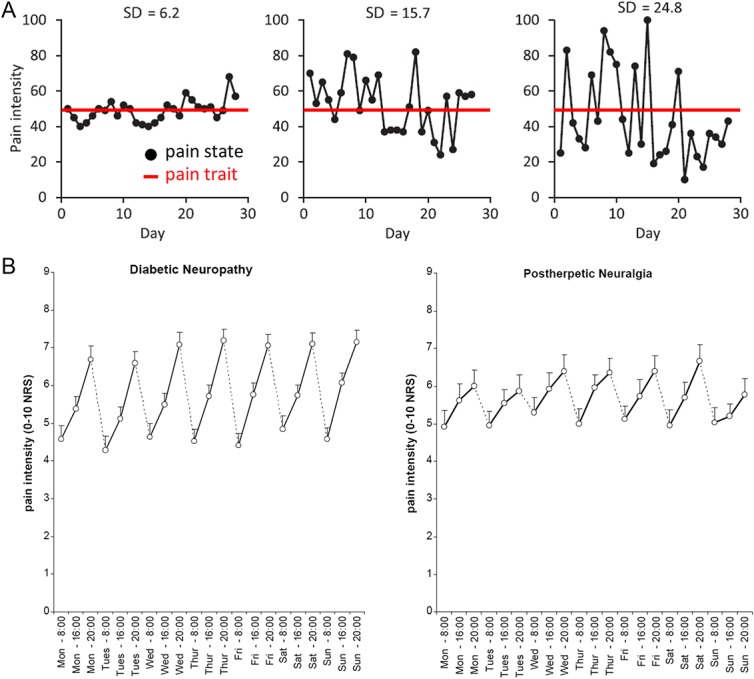
Fluctuations in chronic pain. (A) Daily fluctuations in ratings of chronic pain in 3 patients with chronic pain are shown in this example and illustrate cases in which patients exhibit different degrees of day-to-day variability in their pain experience, yet exhibit a similar overall mean level of pain as assessed over 1 month. Daily pain measures represent state pain, and the mean level of pain assessed over a month represents trait pain (modified with permission^36^). (B) Circadian patterns of pain in patients with diabetic neuropathy (left) and postherpetic neuralgia (right) illustrate how pain varies over time (with permission^32^). NRS, numerical rating scale; dplns, dorsal posterior insula.

In chronic pain conditions, patients can exhibit stimulus-evoked pains (ie, allodynia and hyperalgesia), and these too can be characterized as traits (ie, a typical response) or states (ie, momentary pain) as described above for acute pain. However, the issue of ongoing (spontaneous) pain is arguably trickier to classify. In addition to the factors that can modify stimulus-evoked pains (attention, arousal, mood, etc), there may be additional modifying conditions that may vary over time (eg, medications, comorbidities, disease progression, etc). In addition, chronic pain can fluctuate on many timescales (moment to moment, hourly, daily, etc), which can be captured in assessments of chronic pain that probe about pain across multiple timescales. Thus, questions about current pain (eg, how much pain do you have now?) provide insight into pain states (ie, momentary pain). Collecting these measures has been facilitated with the advent of smartphone apps over the past few years, which can be used to obtain and track pain ratings over time. But, to assess pain trait requires a patient to reflect on their average or typical pain over a longer period (eg, a week or month). Some patients may exhibit a stable level of pain, while other patients may experience highly variable pain from day to day; yet, these different patients may report a similar overall average pain (trait) over time^36^ (see examples in Fig. 2). It is also interesting to consider that the degree of fluctuations in pain ratings not only represents different states but also a trait that characterizes a variability factor. This highlights the importance of investigating both pain trait and states, not only to help guide treatment but also to inform the interpretation of research outcomes (see below). Finally, we note that a patient's typical (trait) pain and momentary (state) pain are not necessarily independent but rather likely influence each other.

Beyond measures of pain per se, it is also important to understand how brain measures are related to measures of daily functioning in chronic pain. As activity levels and function can similarly exhibit day-to-day variability, it would be insightful to determine not only how brain measures are related to measures of function derived from a single point in time but also the average over a period. Towards this goal, it has been suggested that objective real-time monitoring such as the use of actigraphy may be able to provide information on these dynamic changes in functioning over time.^40^

## 4. How are pain states and pain trait represented in the brain?

Brain imaging can provide information about 3 major organizational components of the brain: structure (gray matter and white matter), function, and connectivity (structural and functional).^11^ One might intuitively assume that measures of brain function (eg, functional magnetic resonance imaging [fMRI], magnetoencephalography (MEG), EEG, and positron emission tomography (PET)) reflect situational brain activity on millisecond to tens of second timescales and thus represent a “pain state.” Conversely, measures of brain structure are more likely to be more stable over time (at least on timescales longer than hours) and thus a better reflection of “trait.” However, these assumptions may be too simplistic and do not account for the complexity and capacity of plasticity across different timescales. For example, it is now well established that there is pronounced gray and white matter plasticity due to learning.^45^

Resting state regional brain activity may show fluctuations within an individual, and in some cases, this could reflect a particular behavioural or pain state. However, it is also possible that the dynamics of regional activity (eg, as reflected by BOLD variability, amplitude of low frequency fluctuations, and spectral frequency EEG/MEG oscillatory measures) reflect a trait as well. This concept has been developed in other fields such as cognition and aging,^17^ and we have discussed this in relation to acute^34^ and chronic pain.^4,23,35^

Functional connectivity was originally conceptualized and calculated as a static snapshot of the synchrony between the signal time series of 2 brain areas over many minutes. However, it was then realized that this synchrony was not always fixed, and that there were significant dynamics of the synchrony over shorter timescales of milliseconds–seconds—known as dynamic functional connectivity.^22^ So, is inter-regional functional connectivity a state or a trait? The answer is likely both—since it can sometimes be stable within an individual,^21^ but at other times can vary according to mental state or other conditions.^18^

What about the brain responses to a noxious stimulus? In general, if experimental conditions are held stable, and the stimulus evoked a consistent level of pain, then the resultant brain responses to multiple stimuli tend to be similar within an individual—ie, a trait response. However, if the experimental conditions are not held constant, then an individual can exhibit a wide range of brain responses to a noxious stimulus—ie, a state response. Of course, these state and trait responses could also show interaction effects, akin to trait–context interactions present in other systems (eg, see discussions in Refs. 16, 29).

## 5. How is imaging used to examine pain?

There are 2 general approaches that have been used to link brain imaging findings to pain.^11^ The most common and simplest approach is to simply correlate brain activity with the stimulus intensity delivered to evoke pain (stimulus-evoked response) or to ratings of some attribute of the evoked pain (intensity, unpleasantness, etc). The pain ratings are obtained either in a separate psychophysical session or at the end of the imaging session—thus representing an overall evaluation of “average pain” during the experiment. From the first fMRI studies of pain, it was clear that same stimulus intensity could evoke different pain experiences (in intensity and quality) upon repeated trials and across individuals,^10,14,15^ and so, a second approach, known as percept-related fMRI, was developed to closely link the magnitude and moment-by-moment time-varying characteristics of specific pain percepts that are evoked by a noxious stimulus over time. This approach was used to discern neural representations of different types of pains such as prickle,^12^ paradoxical heat,^13^ rectal pain,^25^ mechanical pain,^27^ and low back pain^2^ and also used to track capsaicin-induced pain intensity using arterial spin labeling.^37^

**Figure 3.**
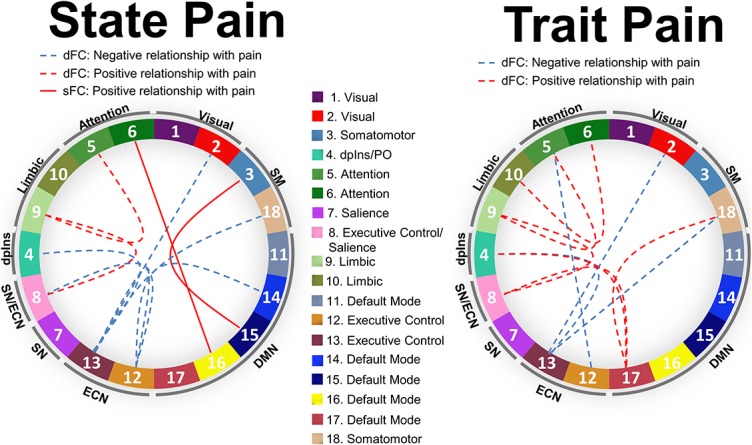
Multivariate brain functional connectivity models for state and trait pain. Displayed for each model are the top-10 most important brain features (largest multivariate weights) in the model for state and trait pain derived from resting state functional connectivity data and pain intensity ratings in 71 patients with chronic neuropathic pain. dFC, dynamic functional connectivity; sFC, static functional connectivity (modified with permission^6^); dplns, dorsal posterior insula; PO, parietal operculum.

Although much was gleaned about the neural representation of pain from the first wave of neural imaging studies, advances in the field have stagnated of late, in part because of the limitations of univariate statistical approaches. However, there are now more sophisticated multivariate and machine learning methods^24,43^ being used to link the brain and pain^26,28,42^ (also see Ref. 31, Necka et al. in this special issue). There are numerous advantages of this more complex approach, but the interpretation of the findings needs to consider whether the study captured/modeled a pain state or trait. Approaches to identify brain states are now being used to examine pain brain states, which include those based on dynamic functional connectivity, such as the hidden Markov model, or K-means clustering of dynamic correlations estimated by sliding windows or dynamic conditional correlation.^1,7,33^ Incorporating knowledge about states is important to inform studies of chronic pain where patients can experience fluctuating pain levels from moment to moment, and day to day as noted above. An illustrative example is the machine learning models we derived from dynamic and static resting state functional connectivity data in patients with neuropathic pain based on either ratings of current pain (state pain) or average pain over a month (trait pain). The features of both the state pain and trait pain models were dominated by dynamic functional connectivity and shared many commonalities but were distinct models (Fig. 3). For example, cross-network dynamic functional connectivity between the default mode network and other brain networks was positively correlated with trait pain, which was not present in the brain model for state pain.

## 6. Importance of linking brain measures with state pain and trait pain

Brain imaging is often used to gain insight into particular behaviours, sensations, and percepts. In the pain field, brain imaging data are often correlated with a general level of pain or specific attribute of pain. It is important for brain imagers to be cognizant of the attribute(s) of pain represented by their data because the pain experience comprises a basic “ouch” sensation as well as sensory-discriminative, motivational-affective, and cognitive-evaluative components (for a discussion of the pain switch model for “ouch,” see Ref. 9). It is also important for imagers to control for or at least be aware of as many of the confounds and factors that impact their data that arise from the context of the experiment and subject conditions.^30^

Brain imaging studies of chronic pain should also consider the issue of state vs trait pain. This is important from a technical and biological viewpoint, and has neuroethical implications. Broadly speaking, the question is how to frame the brain imaging findings; do they represent how the individual is feeling at the moment of the scan window (pain state) or their more general condition (pain trait)? Chronic pains can fluctuate over timescales of minutes to hours to days because of many factors (activity, arousal, medications, fatigue, circadian effects, etc). For example, a patient with chronic pain may be having a particularly “good” or “bad” day at the time of brain imaging acquisition, and so, the findings may not be reflective of their “typical” pain experience. How we interpret such findings is critical to build an accurate view of the “chronic pain brain.” Furthermore, understanding a state pain brain vs a trait pain brain is also important to discern to accurately inform treatment plans and diagnostics, and has obvious neuroethical implications.^8^

## 7. Pros and cons of linking the brain to pain state vs pain trait

Studies of pain states and pain trait provide complementary information, each having advantages and limitations (Table 1).

**Table 1:**
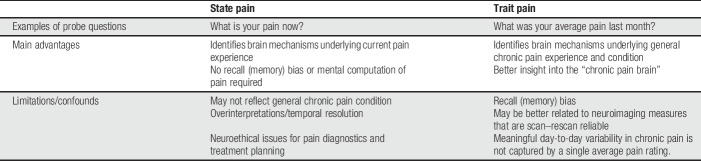
Linking brain measures with state pain and trait pain.

The main advantage of state pain studies is that they can provide insight into how the brain represents the pain experience as it occurs. Another advantage is that pain ratings provided at the time of a scan (or during the scan itself) do not require the subject to recall their pain experience from memory or to perform some sort of calculation of how they generally felt over time. However, unless a percept-related approach is used with continuous ratings, the timing of the ratings (eg, before/after the scan) and the temporal resolution of the imaging modality may not provide the granularity to precisely match a percept with brain activity, and so, caution must be used to avoid overinterpretation of the findings. As noted earlier in this review, there are a host of situational factors that can impact the pain experience (attention, mediations, alertness, etc), and so, another limitation of state pain studies is that they may not reflect the typical or average chronic pain condition experienced by a patient. Finally, there are neuroethical issues associated with the use of state pain data because they may not reflect well the general “brain in chronic pain.” For example, these brain scans may result in false negatives or false positives that would have deleterious consequences for chronic pain diagnostics, insurance claims, and personalized pain management decisions.

Studies of trait pain also have both utility and limitations. This type of study can provide insight into mechanisms underlying the general pain condition of the patient. It also may better represent the pain that a patient is generally experiencing and thus may be more relevant than a state pain study to gain insight into the overall brain abnormalities that drive or maintain chronic pain in that patient. One confound of trait pain measures is the dependence on recalling how much pain has been experienced over a period can introduce biases and may be inaccurate.^3^ It may be particularly challenging for a patient to provide an average pain score if their pain fluctuates greatly over time. One method to alleviate this confound is the use of pain diaries. Although the use of paper diaries can be impacted by poor compliance or backfilling,^39,44^ electronic diaries which can time and date-stamp each entry for validation can reduce this problem.^36^ There is also no agreed upon period (eg, 1 week and 1 month) to use to assess trait pain. Finally, an alternate approach to understand the brain representation of pain trait is to identify stable brain characteristics present in multiple brain scans acquired over weeks or months.

## Disclosures

The authors have no conflict of interest to declare.
